# *Lactobacillus johnsonii* YH1136 plays a protective role against endogenous pathogenic bacteria induced intestinal dysfunction by reconstructing gut microbiota in mice exposed at high altitude

**DOI:** 10.3389/fimmu.2022.1007737

**Published:** 2022-10-10

**Authors:** Zhiqiang Wan, Xufei Zhang, Xianhao Jia, Yuhua Qin, Ning Sun, Jinge Xin, Yan Zeng, Bo Jing, Jing Fang, Kangcheng Pan, Dong Zeng, Yang Bai, Hesong Wang, Hailin Ma, Xueqin Ni

**Affiliations:** ^1^ Animal Microecology Institute, College of Veterinary, Sichuan Agricultural University, Chengdu, China; ^2^ Plateau Brain Science Research Center, Tibet University, Lhasa, China; ^3^ College of Basic Medical Sciences, Chengdu University of Traditional Chinese Medicine, Chengdu, China; ^4^ Guangdong Provincial Key Laboratory of Gastroenterology, Department of Gastroenterology, Institute of Gastroenterology of Guangdong Province, Nanfang Hospital, Southern Medical University, Guangzhou, China; ^5^ Guangzhou Beneco Biotechnology Co. Ltd., Guangzhou, China

**Keywords:** high-altitude exposure, probiotic, *Lactobacillus johnsonii*, intestinal microbiota, miRNA

## Abstract

**Background:**

Intestinal microbiota plays an important role in maintaining the microecological balance of the gastrointestinal tract in various animals. Disturbances in the intestinal microbiota may lead to the proliferation of potentially pathogenic bacteria that become the dominant species, leading to intestinal immune disorders, intestinal inflammation, and other intestinal diseases. Numerous studies have been confirmed that high-altitude exposure affects the normal function of the intestine and the composition of the intestinal microbiota. However, it is still necessary to reveal the changes in intestinal microbiota in high-altitude exposure environments, and clarify the relationship between the proliferation of potentially pathogenic bacteria and intestinal injury in this environment. In addition, explored probiotics that may have preventive effects against intestinal diseases.

**Methods and results:**

C57BL/6 mice were randomly divided into three groups, a high-altitude group (HA), control group (C), and high-altitude probiotic group (HAP). The HA and HAP groups were subjected to hypoxia modeling for 14 days in a low-pressure oxygen chamber with daily gavage of 0.2 mL of normal saline (HA) and *Lactobacillus johnsonii* YH1136 bacterial fluid (HAP), while the control group was fed normally. *L. johnsonii* YH1136 was isolated from feces of a healthy Tibetan girl in Baingoin county, the Nagqu region of the Tibet Autonomous Region, at an altitude of 5000 meters. Our observations revealed that gavage of YH1136 was effective in improving the damage to the intestinal barrier caused by high-altitude exposure to hypoxic environments and helped to reduce the likelihood of pathogenic bacteria infection through the intestinal barrier. It also positively regulates the intestinal microbiota to the extent of Lactobacillus being the dominant microbiome and reducing the number of pathogenic bacteria. By analyzing the expression profile of ileal microRNAs and correlation analysis with intestinal microbiota, we found that Staphylococcus and Corynebacterium1 cooperated with miR-196a-1-3p and miR-3060-3p, respectively, to play a regulatory role in the process of high-altitude hypoxia-induced intestinal injury.

**Conclusion:**

These findings revealed the beneficial effect of *L. johnsonii* YH1136 in preventing potential endogenous pathogenic bacteria-induced intestinal dysfunction in high-altitude environments. The mechanism may be related to the regulation of intestinal injury from the perspective of the gut microbiota as well as miRNAs.

## Introduction

Probiotics can maintain the stability of intestinal microbiota in the body, which is of great significance to the health of humans and animals (1). In recent years, there has been an increasing understanding of the use of probiotics for the prevention and treatment of intestinal diseases caused by pathogenic bacteria or intestinal immune dysregulation; however, there is a lack of understanding that exists in high-altitude exposure environments. The features of high-altitude exposure are low air pressure and hypoxia, which profoundly affect the physiological function and status of organs and are considered serious problems (2, 3).Studies have shown that intestinal microbiota disorders are closely related to impaired intestinal function, and acute high-altitude exposure leads to intestinal microbiota disorders that affect intestinal barrier function, which in turn may cause bacterial translocation and, consequently, intestinal inflammation (4–6). These are also considered to be important potential factors in the progression of inflammatory bowel disease (IBD) (6–8), but the mechanisms by which high-altitude hypoxic environments affect intestinal function are not yet clear. Disturbances in the intestinal microbiota can lead to a predominance of pathogenic bacteria in the gut, resulting in intestinal infections. Pan et al. (9) reported that the gut microbiota in a hypoxic environment appeared different, characterized by an increased abundance of the genera *Parabacteroides*, *Alistipes* and *Lactococcus* and an increased ratio of *Bacteroides* to *Prevotella*. In addition, disruption of the gut microbiota increases susceptibility to infection (10), and the intestinal microbiota usually plays an important role in immune regulation in maintaining host immune homeostasis and maintaining intestinal health together with the intestinal barrier (11). Previous studies have revealed that a special living environment makes the gut microbiota of animals in high-altitude areas unique facilitating their adaptation to high-altitude environments (12, 13). This suggests that altitude-related intestinal issues can be investigated from the perspective of the intestinal microbiota. A novel approach to coping with intestinal injury induced by a high-altitude hypoxic environment may be the use of probiotics to modulate the composition of the intestinal microbiota.

However, few studies have examined the effects of probiotics in plateau environment. Based on previous studies, we hypothesized that sudden exposure to a plateau environment disrupts the intestinal microbial balance, where pathogenic bacteria that become dominant species may disrupt intestinal immunity leading to intestinal inflammation, and that probiotic supplementation may have a regulatory effect on this process. We intend to use *L. johnsonii* YH1136 in further studies. *Lactobacillus* spp. is a widely used probiotic. We isolated *Lactobacillus johnsonii* YH1136 (CCTCC M 20221116) from feces of a healthy Tibetan girl in Baingoin county, the Nagqu region of the Tibet Autonomous Region, at an altitude of 5000 meters. Based on our previous studies, *Lactobacillus johnsonii* shows promising results by improving intestinal microbiota in preventing and treating nonalcoholic fatty liver disease, modulating stress and memory impairment based on the gut-brain axis, and plays an important regulatory role in processes involved in renal and intestinal injury induced by high fluoride exposure (10, 14–19). Therefore, this study evaluated whether *L.johnsonii* YH1136 is effective in mitigating damage to intestinal integrity due to hypoxic environments in rats exposed to high altitudes. In addition to the intestinal microbiota, microRNAs (miRNAs) also play regulatory role during intestinal injury. It has been reported that miRNAs can regulate proteins involved in intestinal barrier integrity and permeability, and are considered biomarkers of some intestinal diseases (20–22). Therefore, we detected and analyzed the expression of miRNAs in the ileum and the composition characteristics of intestinal microbiota concurrently, in order to describe the changes in the intestinal environment after high-altitude hypoxic stress and explore the preventive mechanism of *L. johnsonii* YH1136.

## Materials and methods

### Animal and experiment design

A total of 36 C57BL/6 mice of the same age and similar growth conditions (8 weeks old, purchased from Chengdu Dashuo Institute of Biology, China) were randomly selected and, divided into three groups and given a week to adapt to their new environment. The three groups were the control (C), high altitude (HA), and high-altitude probiotic (HAP) groups. The air pressure level for group C was maintained at 94.5 kPa, while the HA and HAP groups were exposed to a low-pressure oxygen chamber simulating an altitude of 3500-4000m for 14 days, with the air pressure in the chamber set to 60-65 kPa. Mice in the HAP group were given 0.2mL *L. johnsonii* YH1136 by gavage every day, while mice in the C and HA groups were given 0.2mL physiological saline (pH 7.0) by gavage as a substitute. The temperature and humidity of the low-pressure oxygen chamber were identical to those outside the chamber. The chamber was opened for one hour per day to supply food (Chengdu Dashuo Institute of Biology, China) and water. All animal experiments were conducted in accordance with the guidelines for the feeding and use of experimental animals (approval no: SYXKchuan2019-187) approved by the Committee of Sichuan Agriculture University. All animals were raised in the animal room of the Animal Microecology Research Institute of Sichuan Agriculture University, with a 12-hour diurnal cycle. The experiment lasted 14 days.

### Sample collection

On day 15, six mice from each group were randomly selected and subjected to cervical dislocation. The ileal segment of mice (approximately 0.5 cm) was collected, washed with cold diethylpyrocarbonate water, and stored in liquid nitrogen until further miRNA analysis. The serum was collected by centrifuging the blood of mice and storing it at -80°C. The ileum was collected from each group, fixed in 4% paraformaldehyde solution, and stored at 4°C for immunohistochemical analysis.

### 16S rRNA gene amplicon sequencing and analysis

PCR amplification of the bacterial 16S rRNA genes V3–V4 region was performed using the forward primer 338F (5’-ACTCCTACGGGAGGCAGCAG-3’) and reverse primer 806R (5’-GGACTACHVGGGTWTCTAAT-3’). The PCR components contained 5μL of buffer (5×), 0.25μL of Fast Pfu DNA Polymerase (5U/μL), 2μL (2.5 mM) of dNTPs, 1μL (10 μM) of each forward and reverse primer, 1 μL of DNA template, and 14.75 μL of ddH2O. Thermal cycling consisted of initial denaturation at 98°C for 5 min, followed by 25 cycles consisting of denaturation at 98°C for 30 s, annealing at 53°C for 30 s, and extension at 72°C for 45 s, with a final extension of 5 min at 72°C. PCR amplicons were purified using Vazyme VAHTSTM DNA Clean Beads (Vazyme, Nanjing, China) and quantified using the Quant-iT PicoGreen dsDNA Assay Kit (Invitrogen, Carlsbad, CA, USA). After the individual quantification step, amplicons were pooled in equal amounts, and pair-end 2× 250 bp sequencing was performed using the Illlumina MiSeq platform with MiSeq Reagent Kit v3 at Shanghai Personal Biotechnology Co., Ltd (Shanghai, China). Bioinformatics analysis was performed using QIIME2 2020.11 (https://docs.qiime2.org/2020.11/tutorials/) and the (V3.1.2)

### Serum biochemical test and statistical analysis

A mouse-specific ELISA kit was used to detect the levels of serum diamine oxidase (DAO) and D-lactate. Statistical analysis of DAO and D-lactate levels was performed by one-way analysis of variance (ANOVA) using IBM SPSS Statistics version 27, to detect the normality and statistical significance of the data. The data was considered to confirm to a normal distribution if the significance value obtained from the Shapiro-Wilk test was > 0.05. One-way ANOVA was used for analysis, and least significant difference (LSD) and Dunnett T3 tests were used for multiple comparisons. Statistical significance was set at P <0.05.

### Immunohistochemical and immunofluorescence analysis

The fixed tissues were flushed with water, dehydrated, made transparent, waxed, embedded, and sectioned to prepare paraffin sections. The sections were placed in citric acid antigen retrieval buffer (pH 6.0) in a microwave oven and incubated after boiling for antigen retrieval. The sections were thereafter placed in a 3% H_2_O_2_ solution and incubated for 25 min in the dark at room temperature, after which the sections were placed in Phosphate-buffered saline (PBS, pH 7.4) and shaken three times for 5 min each on a shaker. The tissue was thereafter uniformly covered and blocked with 3% bovine serum albumin (BSA) for 30 min at room temperature. The blocking solution was gently discarded, and rabbit polyclonal anti-occludin (1:200), rabbit polyclonal anti-claudin-1 (1:200), rabbit polyclonal anti-ZO-1 (1:200), rabbit polyclonal anti-IL-1β (1:200), rabbit polyclonal anti-TGF-β (1:200), and rabbit polyclonal anti-TNF-α (1:200) antibodies were sequentially added to the sections and incubated overnight at 4°C. The antibody solutions (occludin, Claudin-1 and ZO-1) were removed by washing with PBS, and Horseradish (HRP)-labeled goat antirabbit IgG (1:200) was added dropwise and incubated for 50 min at room temperature. Finally, the cells and nuclei were stained using the immunohistochemical DAB chromogen kit and hematoxylin, respectively. Sections for inflammatory factors (occludin, claudin, and ZO-1) were counterstained for nuclei with DAPI; an autofluorescence quencher was added, and an anti-fade mounting medium was used to block the slides. The prepared sections were placed under a fluorescence microscope for observation and to obtain images. All above reagents were purchased from Servicebio Technology Co., Ltd. (Wuhan, China).

### Small RNA library preparation and sequencing

Total RNA was extracted from each group using a Total RNA Isolation Kit (Omega Inc., Norcross, Georgia, United States) according to the manufacturer’s instructions. The library was constructed and sequenced as follows. First, an Agilent 2100 Bioanalyzer was used to detect the RNA concentration and integrity. The TruSeq Small RNA Sample Prep Kit was used to construct a small RNA library. Next, the library was enriched by PCR amplification, and the sequencing adaptor and index parts were added. The library was thereafer purified by gel electrophoresis. An Agilent 2100 Bioanalyzer was used to test the quality of the library with an Agilent High Sensitivity Kit, where the qualified library should have a single peak. The the library was quantified using a Quant-iT PicoGreen dsDNA Assay Kit, and finally, an appropriate loading amount was selected and sequenced on an Illumina platform.

### Analysis and identification of microRNA

The raw data was processed to remove the adaptor sequences and filter the unqualified data. We then merged identical sequences and recorded the richness of the remaining sequences. Based on the mouse reference genome, the processed data were annotated and compared to obtain relevant information on small RNA. Small RNA includes miRNAs, piwi-interacting RNA, and non-coding RNAs (ncRNAs). Subsequent analysis was performed mainly on the miRNAs. We downloaded the sequences of mouse precursors and matures miRNA form miRBase, and then compared unique reads with the downloaded sequences to annotate the sequencing results. According to the comparison results, we used mireap to analyze the sequences that have not yet been annotated, and predicted novo miRNAs.

### Analysis of known miRNA expression

The read counts of the measured miRNAs were calculated according to the sequence number of mature miRNAs in mice. Because a miRNA may have multiple precursors that produce potentially identical mature miRNAs, we chose to name the abundance of the first occurrence of the same name miRNAs as the abundance of the miRNAs for subsequent analysis.

### miRNA target prediction and functional analysis

miRNAs mainly bind to target sites through complementary pairing. We used the miRanda software to predict the target genes of the differentially expressed miRNAs. Using the 3- ‘UTR’ of mouse mRNA as the target sequence, the number of target genes and target sites of differentially expressed miRNAs were predicted. We then used the topGO software for enrichment analysis, where we used Gene Ontology (GO) terms to annotate the different miRNA target genes and calculate the number of miRNA target genes of each term in order to determine which of the different miRNA target genes were significantly enriched in the whole genomic context (P-value < 0.05 indicated significant enrichment). Thus, the main biological functions of differentially expressed miRNA target genes were determined.

### The validation of differential expression of miRNA by RT-qPCR

We used the stem-loop RT-qPCR method (23) to verify the differential expression of miRNAs and randomly selected differentially expressed miRNAs from each of the two groups were balanced. Total RNA was extracted using a Total RNA Isolation Kit (Omega Bio-Tek, Norcross, GA, USA) according to the manufacturer’s instructions. The purity and concentration of the RNA were assesed using a NanoDrop 2000 spectrophotometer (Thermo Fisher Scientific Inc., Waltham, MA, USA), and cDNA was generated by reverse transcription using the M-MLV 4 First-Strand cDNA Synthesis Kit (Biomed, Beijing, China). The expression of miRNAs from the cDNA was detected using SYBR Green real-time fluorescence quantitative PCR. Stem-loop RT-qPCR is a common method used for detecting miRNA expression. It is characterized by the use of special primers with a stem structure to reverse transcribe RNA in the reverse transcription stage. Specific primers were designed to combine the products for subsequent fluorescence quantitative detection experiments. The reaction volume was 10 µL, which including 5 µL SYBR Green Master Mix (Bio-Rad),1 µL forward primer, 1µL reverse primer, and 100ng cDNA. The remained was supplemented to 10 µL with RNase-free water. The reaction was conducted on a LightCycler ^®^96 System (Roche, Germany). The programs for real time qPCR reactions were as follows: pre-incubation at 95°C for 300 s, 40 cycles of 95°C denaturation for 10s and 60°C annealing for 30s. Final melting curve analysis was performed to determine the purity of the PCR products. The specific primers for RT-PCR is shown in **Table S1** and the Primers for qPCR is shown in **Table S2**.

### Correlation analysis between gut microbiota and miRNAs

We quantified changes in community networks underlying microbial associations among the three groups using Netshift analysis, identifying microbes that may have a driving role for some of them.

To identify the crucial microbial species for high-altitude exposure and probiotic treatment, indicator species analysis was applied to calculate the indicator value of an Amplicon Sequence Variant (ASV) positive specificity and fidelity with one or more group, which was performed with 104 permutations and identified as significant at P < 0.05. Then, Sparcc correlation was used to calculate microbial genera in each group or ASV in all group associations, and a highly significant correlation (ρ > 0.8 and P < 0.01) was considered significant. The greedy optimization of modularity algorithm was applied to select and visualize the community modules in the co-occurrence network. Further, Netshift analysis was applied to explore the changes in the microbial network in the gut community among normal mice, hypoxia-exposed mice, and YH1136-treated mice, from which the genus was found to the main driving force. After these analyses, the crucial microbial genera and key ASVs were integrated to identify the species that could have the greatest effects on the obvious prevention of inflammatory injury induced by long-term hypoxic exposure by gut probiotics. Finally, Spearman’s correlation was used to calculate the degree of association (ρ > 0.8 and P < 0.01) between the selected ASVs and the miRNAs that were significantly different between groups and to visualize their networks and heatmaps.

## Results

### *Lactobacillus johnsonii* YH1136 alleviates intestinal barrier damage and inflammation induced by high-altitude hypoxic stress

**Figure 1:**
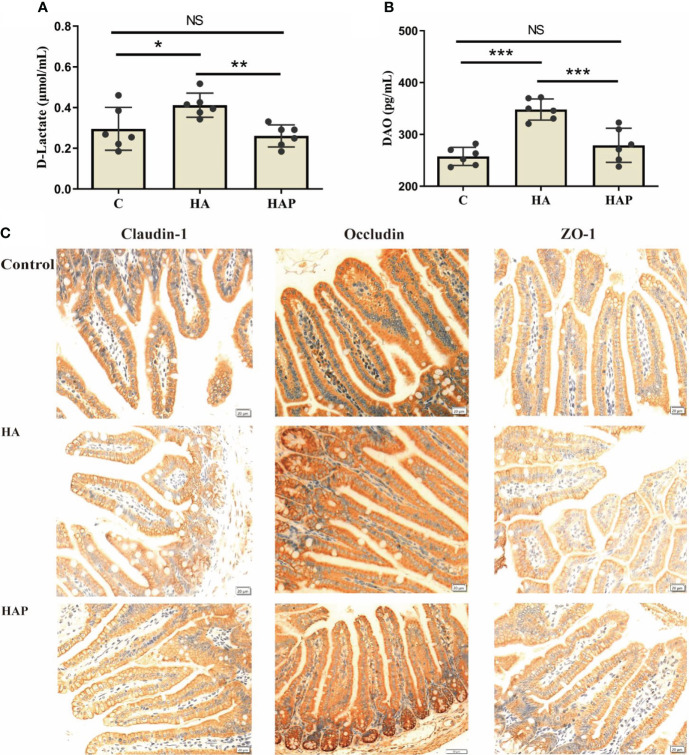
Evaluation and analysis of intestinal permeability **(A)** Serum D-Lactic content. **(B)** Serum DAO content. Data are presented with the meas ± standard deviation (n=6). The marked * between the histograms indicates that the difference is significant. * means *P*<0.05, ** means *P*<0.01, *** means *P*<0.001. **(C)** Immunohistochemical analysis of the expression of mouse ileal tight junction proteins (Claudin-1, Occludin and ZO-1). The positive cells of the three proteins were stained brown. ns, not significant.

**Figure 2:**
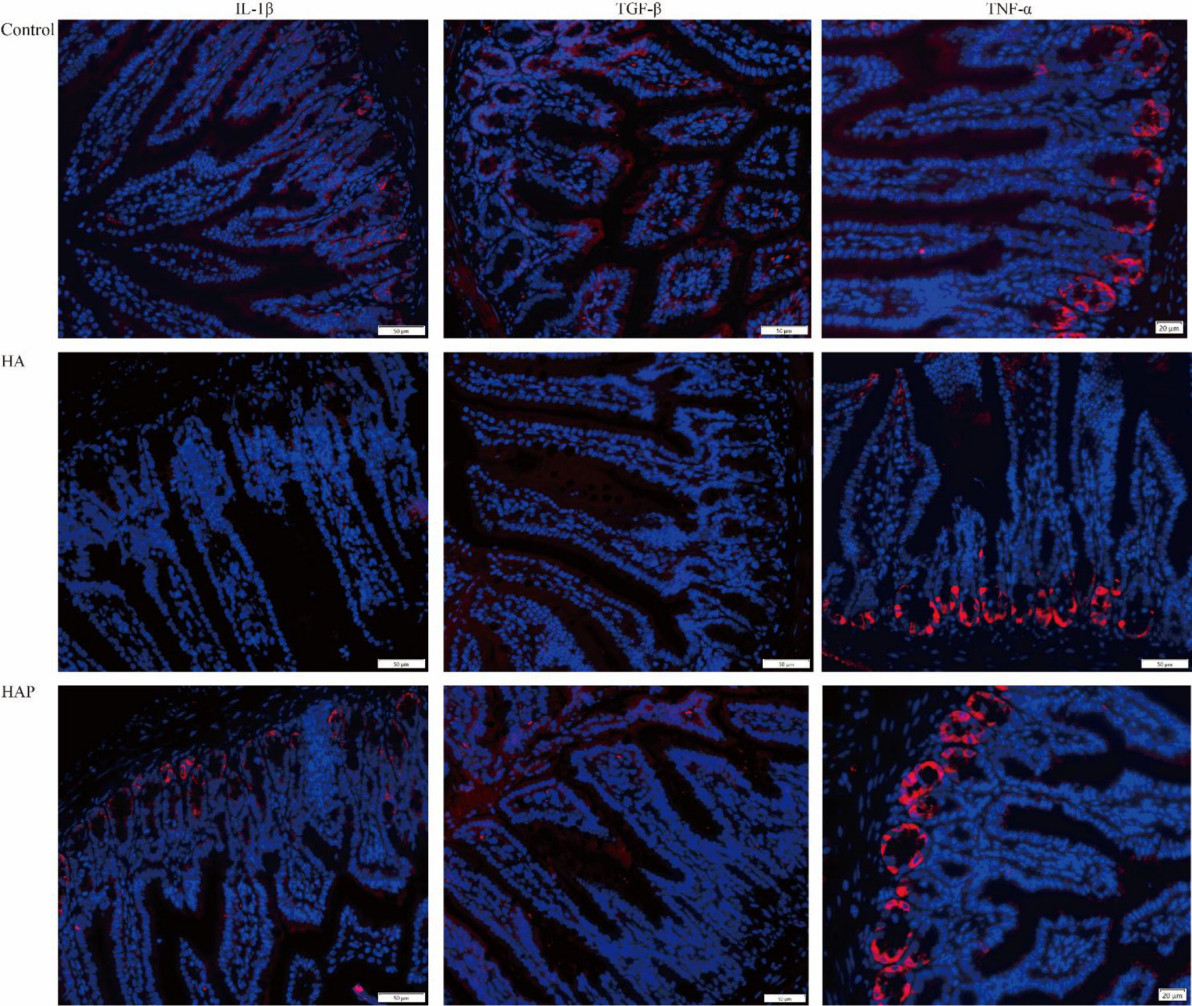
Analysis of inflammation factors in ileum DAPI was used to stain the nucleus, and different fluorescent genes were connected through secondary antibodies to show the expression of the target gene. The target genes were showed in red.

The results of serum enzyme-linked immunosorbent assay (ELISA) showed that the levels of D-lactate and DAO in the HA group were significantly higher than those in groups C and HAP, but there was no significant difference between groups HAP and C (**Figures 1A, B**). Immunohistochemical analysis (**Figure 1C**) of the three tight junction proteins in the HA group decreased (the number of brown positive cells decreased), and supplementation with *L. johnsonii* YH1136 partially alleviated this decreasing trend. DAPI staining showed the expression profiles of IL-1β, TNF-α, and TGF-β, which are inflammatory factors in cells (**Figure 2**). These results suggest that the hypoxic environment at high altitudes may lead to intestinal inflammation and improve intestinal mucosal permeability or damage the intestinal barrier in mice, and that intragastric administration of *L. johnsonii* YH1136 could alleviate these effects to a certain extent.

### *Lactobacillus johnsonii* YH1136 modulates gut microbiota disturbance induced by high-altitude hypoxic stress

**Figure 3:**
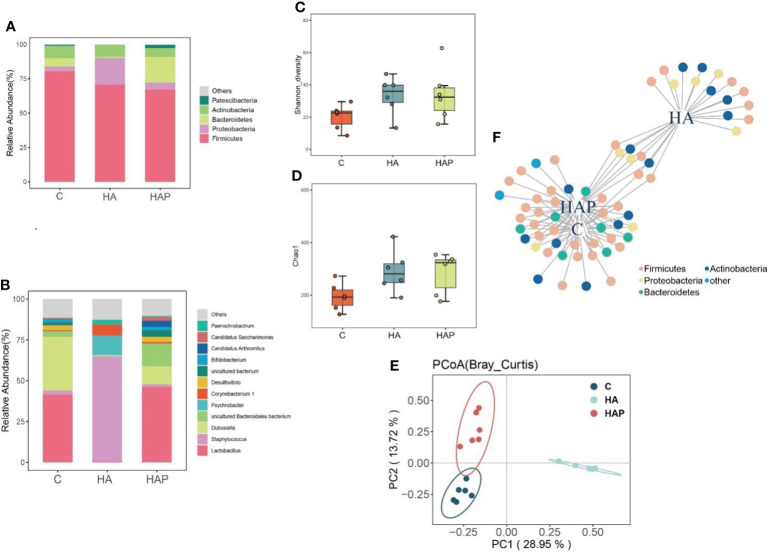
Effects of hypoxia environment and Lactobacillus johnsonii YH1136 supplementation on the composition, structure and diversity of mice ileal micriobiome **(A)** Phylum level species composition. **(B)** Genus level species composition. **(C)** Shannon diversity analysis. **(D)** Chao1 Index **(E)** PCoA (Bray_Curtis) analysis. **(F)** Indicator species analysis, the species sensitive to different treatments are divided into one plant different node colors represent different Phylum levels.

**Figures 3A, B** show the composition analysis of the intestinal microbiota at the phylum and genus levels, respectively. The results of the phylum level analysis (**Figure 3A**) showed that *Firmicutes* were most abundant in the three groups. The abundance of *Firmicutes* was the highest in group C and lower in the remaining two groups. There was a significant increase in the abundance of *Proteobacteria* in the HA group compared to that in group C, whereas the abundance of *Proteobacteria* in the HAP group reverted to that of group C. In addition, *Bacteroidetes* showed distinct differences among the three groups: a substantial decrease in abundance in the HA group and an increase in abundance in the HAP group compared to that in group C. These results suggest that high-altitude exposure may be beneficial to the colonization of *Proteobacteria* and adverse to colonization by *Bacteroides*, which was reversed by *L.johnsonii* YH1136 supplementation. In the analysis of species composition at the genus level (**Figure 3B**), changes in *Staphylococcus* spp. and *Lactobacillus* spp. were of the greatest concern. In the high-altitude exposure environment, *Lactobacillus* spp. almost completely disappeared in group HA, replaced by *Staphylococcus* spp., which is a common opportunistic pathogen in mammals (24), and its abundance sharply increased from less than 5% in group C to > 60% in group HA. In the HAP group, *Lactobacillus* abundance was effectively reversed after supplementation with *L.johnsonii* YH1136.

As shown in **Figures 3C, D**, the Shannon diversity and Chao1 index of groups HA and HAP were higher than those of the control group, and there was no significant difference between the other groups. Principal coordinate analysis (**Figure 3E**) showed that the distance between group HAP and group C was relatively close, indicating that the structure of intestinal microbiota in group HAP was the most similar to that in group C; the distance between groups HA and C was the farthest, indicating that the similarity of intestinal microbiota structure between group HA and group C was the worst. Indicator species analysis is usually used to explore species differences in intestinal microbiota between groups and the common and representative species of each group. Based on the results shown in **Figure 3F**, we observed that the important indicator species belonged to *Firmicutes*, *Proteobacteria*, *Bacteroidetes*, and *Actinobacteria* under the different treatments. In addition, there were four, fifteen, and five special indicator species in C, HA and HAP groups belonging to *Firmicutes*, *Actinobacteria* and *Proteobacteria*, respectively.

**Figure 4:**
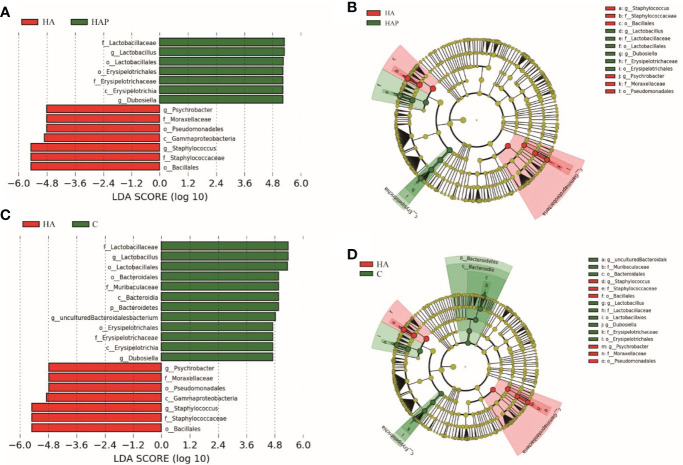
Lefse analysis of ileal microbiota in mice The linar discriminant analysis (LDA) score **(A, C)** and cladogram **(B, D)** were generated from LDA effect size. Taxa with LDA values larger than 4.5 are shown in figure.

LEfSe is an algorithm for the discovery of biomarkers that identify genomic features (genes, pathways, or taxa) that characterize the differences between two or more biological conditions (25). Compared with the HAP group (**Figures 4A, B** **)**, the significant discriminative taxa in group HA were *Psychrobacter*, *Moraxellaceae*, *Pseudomonadales*, *Gammaproteobacteria*, *Staphylococcus*, *Staphylococcaceae*, and *Bacillus*; and in the HAP group, *Lactobacillaceae*, *Lactobacillus*, *Lactobacillales*, *Erysipelottrichales*, *Erysipelotrichaceae*, *Erysipelotrichia*, and *Dubosiella*. Compared with the HA group (**Figures 4C, D**), the significant discriminative taxa in group C were *Lactobacillaceae*, *Lactobacillus*, *Lactobacillales*, *Bacteroidales*, *Muribaculaceae*, *Bacteroidia*, *Bacteroidetes*, *uncultured-Bacteroidalesbacterium*, *Erysipelotrichales*, *Erysipelotrichaceae*, *Erysipelotrichia*, and *Dubosiella*.

### Effects of high-altitude exposure and *Lactobacillus johnsonii* YH1136 supplementation on ileal miRNA expression

The sequencing of all groups yielded more than 15000000 raw reads on average, and the clean read ratio was > 80% in every sample (**Table 1**). We removesd the adapters and filtered the low-quality reads to obtain the clean reads. Clean reads with a sequence length of 18-36nt were counted, and the identical sequences in a single sample were merged to remove the repetitive reads, which are referred to as unique reads (**Figure S1**). There were 185830 unique reads shared by the three groups. Next, we counted the sequence abundance, and the results showed that the length of most unique reads ranged from 18 to 23nt (**Figure S2**). We compared unique reads with the reference genome to obtain the distribution of small RNA in the genome (**Figure S3**). The results showed that small RNAs were distributed in all chromosomes, mainly concentrated in the positive chain of the chromosome 1 reference genome and the negative chain of the chromosome 17 reference genome.

**Table 1:** The ileum small RNA sequencing reads of mice.

Sample	raw reads	clean reads	ratio
HA1_IL	16217114	14251887	87.88%
HA2_IL	16743594	15140637	90.43%
HA3_IL	15637053	13903562	88.91%
HA4_IL	16582976	15239407	91.90%
HA5_IL	20309287	18312484	90.17%
HA6_IL	18368184	16556088	90.13%
C1_IL	17392932	13983541	80.40%
C2_IL	18634960	15382544	82.55%
C3_IL	16611439	14628031	88.06%
C4_IL	19672768	16940003	86.11%
C5_IL	15927969	13826621	86.81%
C6_IL	16653855	13712574	82.34%
HAP1_IL	16678116	14762780	88.52%
HAP2_IL	17107698	15405765	90.05%
HAP3_IL	14754402	12585667	85.30%
HAP4_IL	17580305	15211040	86.52%
HAP5_IL	15159039	13051144	86.09%
HAP6_IL	15362615	13387490	87.14%

**Figure 5:**
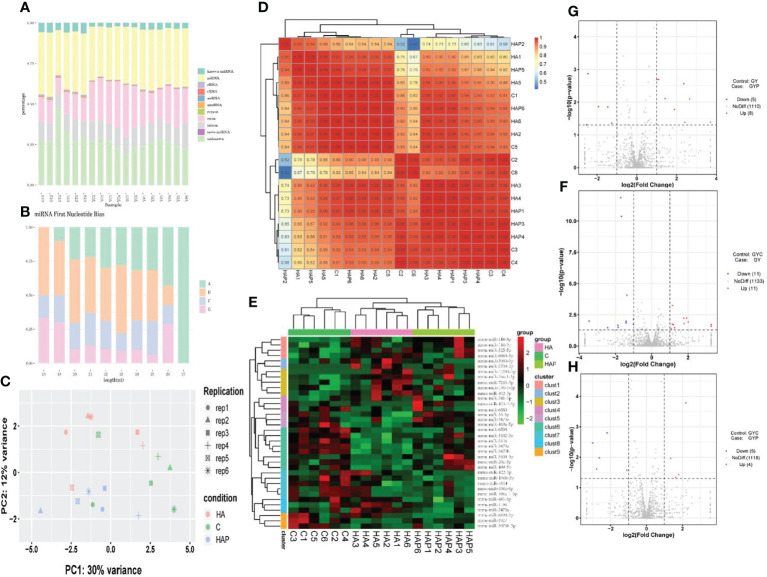
miRNA sequencing analysis **(A)** Unique reads of Small RNA classification statistics. **(B)** miRNA first base preference. **(C)** Principal component analysis of samples in each group. **(D)** Correlation analysis of miRNA expression levels among samples. **(E)** Cluster analysis of differentially expressed miRNAs. **(F–H)** The volcano plot of differentially expressed miRNAs.

By comparing precursor and mature miRNAs of mice in the miRBase database, we annotated the unique reads, and obtained a statistical table of miRNA annotation results (**Table 2**). We then used mireap to perform a new miRNA prediction analysis on sequences that were not annotated; the results are shown in **Table 3**. In this way, we identified 147, 149, and 127 miRNAs in the HA, C and HAP groups, respectively. We also annotated the classification and proportion of all small RNAs (**Figure 5A**), analyzed the characteristics of all known miRNAs (**Figure 5B**), and performed principal component analysis (PCA) on all samples (**Figure 5C**). Before differential expression analysis, the correlation between miRNA expression levels among the samples was tested to ensure the reliability and rationality of the test (**Figure 5D**). According to the miRNA expression data in each sample, the differential expression of miRNA was analyzed by DEseq (version 1.18.0), and the different conserved miRNAs were screened according to the multiple differences in expression (|log2foldchange| > 1) and the significance of expression differences (p-value < 0.05). A volcano map of differentially expressed miRNAs was drawn in the R statistical environment using the ggplot2 package (**Figures 5F–H**). A total of 44 differentially expressed miRNAs were identified among the three groups. Cluster analysis was used to determine the expression patterns of differentially expressed miRNAs under different experimental conditions (**Figure 5E**). miRNAs with high expression correlations among samples were classified into one category.

**Table 2:** The ileum small RNA sequencing reads of mice.

Sample	miRNA	precursor
HA1_IL	686	538
HA2_IL	693	540
HA3_IL	700	557
HA4_IL	703	549
HA5_IL	775	601
HA6_IL	710	560
C1_IL	706	553
C2_IL	681	535
C3_IL	705	554
C4_IL	745	593
C5_IL	683	552
C6_IL	712	557
HAP1_IL	688	542
HAP2_IL	668	535
HAP3_IL	697	558
HAP4_IL	714	551
HAP5_IL	653	519
HAP6_IL	689	548

**Table 3:** Prediction of novel miRNAs.

sample	novo	unique	total
HA1_IL	9	12	26
HA2_IL	8	11	30
HA3_IL	6	10	16
HA4_IL	11	15	26
HA5_IL	10	12	24
HA6_IL	11	21	25
C1_IL	9	15	26
C2_IL	8	11	16
C3_IL	6	12	22
C4_IL	10	20	38
C5_IL	10	11	20
C6_IL	11	16	27
HAP1_IL	8	10	17
HAP2_IL	8	11	13
HAP3_IL	10	13	19
HAP4_IL	11	15	30
HAP5_IL	6	8	20
HAP6_IL	9	13	28

### Target gene prediction of differentially expressed miRNA and RT-qPCR verification

**Figure 6:**
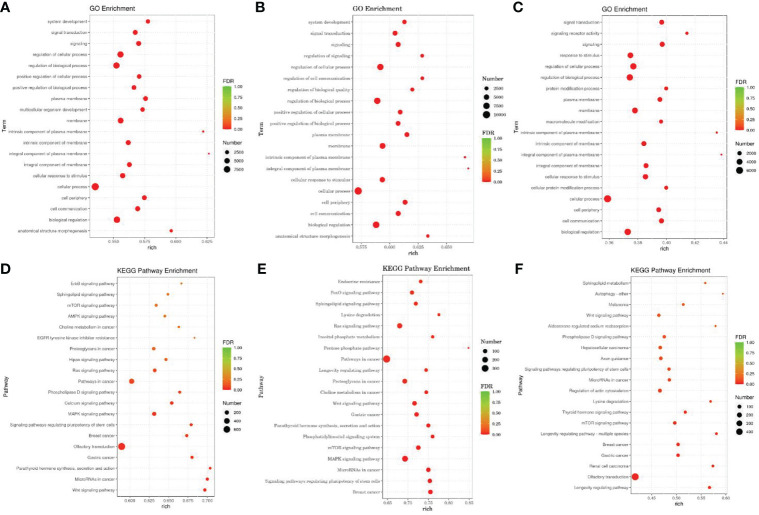
GO and KEGG Pathway enrichment analysis of target genes of differentially expressed miRNAs According to the enrichment results of Go, and the degree of enrichment is measured by rich factor, FDR value and the number of miRNA target genes enriched on this Go term and pathway. The larger the rich factor, the greater the degree of enrichment. The general value range of FDR is 0-1. The closer it is to zero, the more significant the enrichment is. **(A–C)** The GO analysis. **(D–F)** The KEGG Pathway enrichment analysis. The horizontal axis is rich factor, which refers to the ratio of the number of differential miRNA target genes enriched into GO term or pathway to the number of differential miRNA target genes annotated, with a larger value indicating more significant enrichment. The vertical axis is GO term or pathway.

We used miRanda to predict the target genes of differentially expressed miRNA sequences with the 3 ‘UTR’ sequence of the mRNA of the species as the target sequence. TopGo was used for GO enrichment analysis to determine the main biological functions of the differentially expressed miRNA target genes. We used the Kyoto Encyclopedia of Gene and Genomes (KEGG) database to enrich and analyze the functions of the miRNA target genes and their related pathways. **Figures 6** shows the top 20 GO terms (**Figures 6A–C**) and KEGG pathways (**Figures 6D–F**) with the lowest false discovery rate (FDR) value.

GO analysis showed that the differentially expressed miRNA target genes in the HA and HAP groups were mainly enriched in system development, signal transduction, and multicellular organism development, while the differentially expressed miRNA target genes in groups C and HA were mainly enriched in signal regulation, cellular communication regulation, and biomass regulation. The differentially expressed miRNAs target gene in the groups C and HAP weere mainly enriched in signaling receiver activity, cellular protein modification processes, and protein modification processes.

In addition, KEGG pathway analysis showed that the significantly enriched pathways of the target genes of differentially expressed miRNAs were the Wnt signaling pathway, miRNAs in cancer, parathyroid hormone synthesis, secretion and action, lysine degradation, phosphatidylinositol signaling system, inositol phosphate metabolism, lysine degradation, thyroid hormone signaling pathway, and the mTOR signaling pathway.

**Figure 7:**
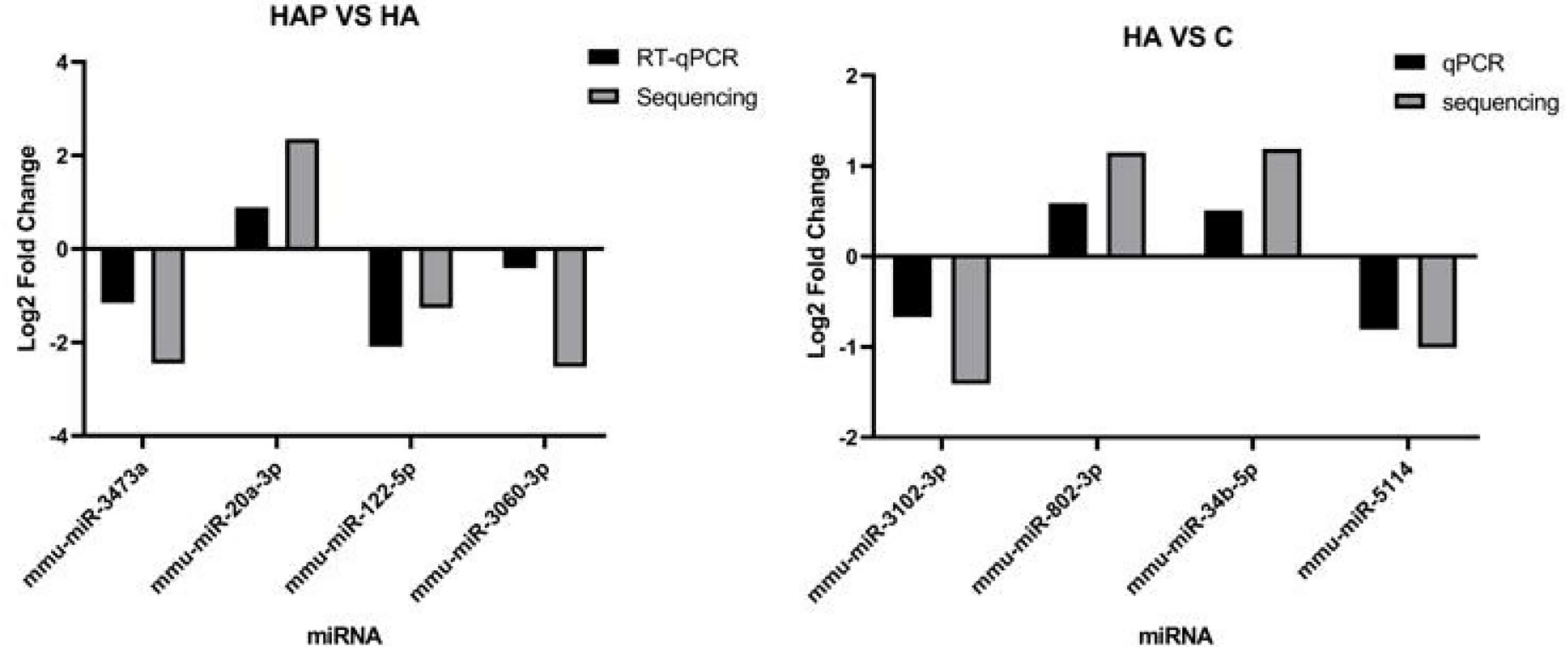
Validation of differentilly expressed of miRNA. RT-qPCR validation experiment of randomly selected differentially expressed miRNAs. Four miRNAs including mmu-miR-3743a, mmu-miR-20a-3p, mmu-miR-122-5p and mmu-miR-3060-3p were selected from HAP group and HA group respectively, and four miRNAs including mmu-miR-3102-3p, mmu-miR-802-3p, mmu-miR-34b-5p and mmu-miR-5114 were selected from HA group and C group to complete the validation test of RT-qPCR.

We used RT-qPCR to analyze miRNA expression and compared it with the sequencing results to determine the accuracy of the results. Four miRNAs with significant differential expression were randomly selected from HA vs. HAP and HA vs. C for quantitative fluorescence analysis. Primer design is presented in **Tables S1, S2**, and the results are shown in **Figure 7**, with U6 snRNA as the housekeeping gene. Fold change was calculated using the △△Ct method and was compared with the results of small RNA sequencing. The results showed that the fold-change trends of the DE miRNAs obtained by the two methods were consistent between groups, and the values were also relatively close, indicating that the sequencing results were reliable.

### The correlation analysis between miRNAs and gut microbiota

**Figure 8:**
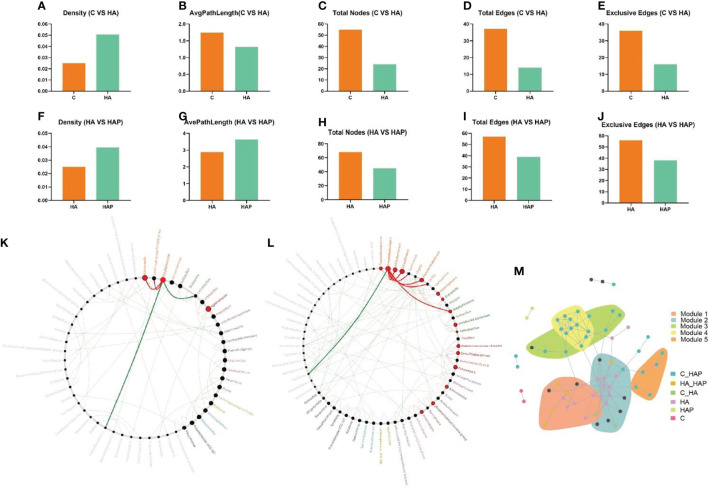
Netshift and co-occurrence network analysis **(A-E)** Changes of network properties between group C and group HA. **(F-J)** Changes of network properties between group HA and group HAP. **(K)** Common sub-networks analysis of group C and group HA. **(L)** Common sub-networks analysis of group HA and group HAP. **(M)** Each node in the co-occurrence network represented an ASV closely related species, which was grouped into the same module, and different colors were used to highlight the response pattern of the ASV module.

In the results of network properties, we noticed an evident decrease in the sub-network average path length, number of nodes, edges, and exclusive edges, as well as an obvious increase in the sub-network density of the microbial community of HA mice compared with the control mice (**Figures 8A–E**). Similarly, with the intake of *L.johnsonii* YH1136, the HAP group reduced the total number of sub-networks of nodes, edges, and exclusive edges; instead, the density and, average path length of the microbial community increased (**Figures 8F–J**).

To explore the driving microbes associated with network changes, we focused on the importance of different microbial genera using the DelBet index and NESH scores. Therefore, nodes (genus) with a positive DelBet index and higher NESH scores in the network shift were observed. The “driver microbes” (top five) that altered the symbiotic network of Genus in the group HA compared to the group C **(** **Table S3** **)** are *Globicatella*, *Staphylococcus*, *Agathobacter*, *Alloprevotella* and *Aminobacterium*. Moreover, compared with group HA **(** **Table S4** **)**, the “driving microbes” (top five) that changed the genus associtation network in the HAP group were *Corynebacterium 1*, *Desulfovibrio*, *Bifidobacterium*, *Pseudochrobactrum*, and *Prevotella 9*.

By integrating the results of the crucial microbial genus and key ASV (**Table S5**), *Staphylococcus* (ASV14, ASV16 and ASV20) and *Corynebacterium 1* (ASV73, ASV60, ASV61 and ASV80) were identified as the driving microbes.

To identify the core modules of the microbial community, we identified five important communities in the co-occurrence network, including indicator species, which corresponded to the response patterns in the species community under hypoxic exposure and *L. johnsonii* YH1136 treatment (**Figure 8M**). We noticed that modules 1 and 2 communities need to be focused on, as most of the species in these module communities had an obvious response to altitude hypoxic environment, but this response disappeared after the supplementation with *L. johnsonii* YH1136. Therefore, the indicator species classified as HA responsive are the key species that we will focus on in further analysis.

**Figure 9:**
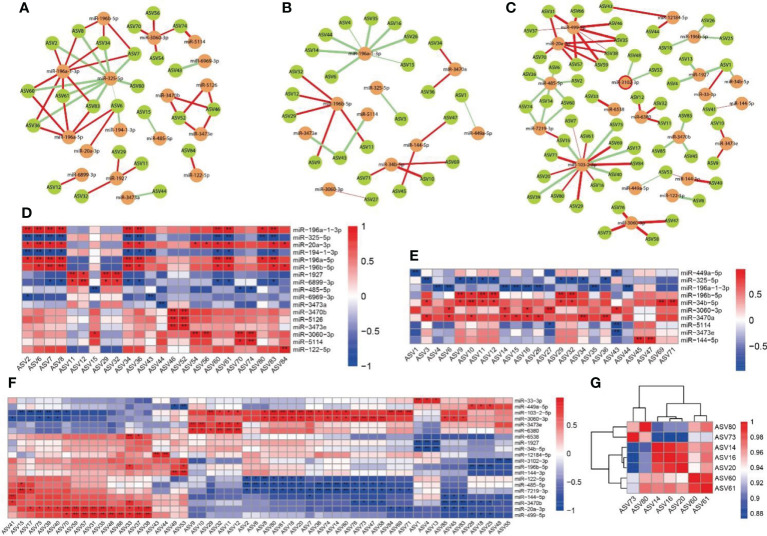
Correlation analysis between miRNAs and intestinal microbiota **(A, D)** Correlations between miRNA in C group and intestinal microbiota. **(B, E)** Correlations between miRNA in HA groups and intestinal microbiota. **(C, F)** Correlations between miRNA in HAP groups and intestinal microbiota. **(G)** Correlation analysis of driving microbes (Staphylococcus and Corynebacterium 1). The colors range form blue to red corresponds to negative correlation and positive correlation, respectively. The ‘*’ means P value < 0.05, ‘**’ means P value < 0.01.

As shown in **Figure 9**, we analyzed the association between differentially expressed miRNAs and intestinal microbiota using the Spearman correlation analysis, and performed a Netshift assay. The results of the Netshift analysis revealed that *Staphylococcus*, which had higher NESH scores in the plateau environment, was a notable gut microbiota driving-species. Additionally, it showed a strong negative correlation with *Lactobacillus*. In the correlation analysis between miRNAs and gut microbiota, miR-196a-1-3p in the HA group had a strong negative correlation with *Staphylococcus*, while miR-3060-3p in the HAP group had a strong positive correlation with *Corynebacterium1*. TargetScan and mirpath were used (26) to predict the target genes and signaling pathways of these two miRNAs **(** **Table S6** **)**. The results showed that miR-196a-1-3p was enriched in ECM-receptor interaction and miR-3060-3p was enriched in the Hippo signaling pathway, which might be the regulatory pathways of high-altitude hypoxic environments and probiotics.

## Discussion

High-altitude areas have lower air pressure and oxygen levels compared to low-altitude areas, which has an impact on the physiological functions of animals and humans, particularly on intestinal microbiota and function (2, 3, 27), inducing gastrointestinal diseases and intestinal microbiota disorders (6, 28, 29). In the past decades of research, changes in the gut microbiota have been increasingly associated with the development of a variety of gastro-intestinal diseases such as inflammatory diseases (8, 30–32). However, the changes in the intestinal microbiota and the regulatory mechanism of probiotics in high-saltitude exposure environment are still unclear. Through 16S high-throughput sequencing analysis, we can understand the changes in intestinal microbiota exposed to high altitude environments, which is helpful in clarifying the resilience of animals and humans to high-altitude exposure environments. In our study, under the simulated hypoxic environments, damage to the intestinal mucosal barrier occurred, with increased permeability and reduced tight junction protein structure. The results of DAPI staining showed that the ileum exhibited inflammatory symptoms in high-altitude exposure environments, and that supplementation with probiotic *L.johnsonii* YH1136 by gavage could alleviate these asymptoms. D-lactate, a metabolite of intestinal bacteria, and DAO produced by intestinal mucosal cells can enter the blood when the intestinal barrier is injured, so the detection of these two indicators in serum can reflect damage to the intestinal barrier (33, 34). In our experiment, probiotic supplementation helped reduce the increased serum D-lactate and DAO content caused by high-altitude modeling, indirectly demonstrating that probiotics played a protective role. This is similar to the results of Khanna et al. (29), on acute hypoxic exposure in rats, which also found varying degrees of intestinal mucosal damage in the form of increased mucosal permeability, and impaired intestinal villus structure. In another study, symbiotic supplementation alleviated intestinal barrier damage induced by hypobaric hypoxic exposure and reduced intestinal inflammation (35).

We found that the composition of the intestinal microbiota in the ileum of mice changed significantly, with *Lactobacillaceae* predominating in group C, whereas *Staphylococcaceae* predominated in the HA group. Meanwhile, the intestinal microbiota of the group HAP gavaged with *L.johnsonii* YH1136 showed a composition similar to that of group C. *Staphylococcus aureus* belongigns to the genus *Staphylococcus* is mostly the pathogenic bacteria, which has been found to be closely related to intestinal infection in lot of studies (36, 37), can cause inflammatory diseases of the intestine, and is a common potentially pathogenic bacterium of the intestine (38, 39). *Lactobacillus salivarius* (40), *Lactobacillus fermentum* (40), *Lactobacillus rhamnosus* (36), and *Lactobacillus kiferi* (41) have been confirmed in several studies to have direct or indirect antibacterial activity against *Staphylococcus aureus*. In our study, the abundance of *Staphylococcus* was significantly increased by simulated high-altitude, which suggesting that the number of pathogenic bacteria in Staphylococcus may also be significantly higher, in other words, a high-altitude hypoxic environment may cause higher numbers of pathogenic bacteria to become the dominant species. But this hypothesis remains to be determined by subsequent analysis. A series of 16S sequencing analysis results revealed that supplementation with *L. johnsonii* YH1136 reversed this trend, returning the gut microbiota at the ileum genus level to the normal *Lactobacillus*-dominated structure.

With the in-depth study of probiotics, there have been several reports on the prevention and treatment of intestinal structural and functional injury (42–44). Small RNA sequencing technology has been continuously developing, and miRNAs are increasingly used in the study of various molecular mechanisms (45, 46). miRNAs are small non-coding RNA, with a length is usually 19-25 nucleotides, which can regulate the post-transcriptional silencing of target genes (47). miRNAs can be involved in regulating a variety of cell activities, including cell differentiation, growth, development, and apoptosis (48). In addition, it has also been demonstrated that hypoxia can alter miRNA expression. Kulshreshtha et al. (49) demonstrated that a specific spectrum of miRNAs (including miR-23, miR-24, miR-26, miR-27, miR-103, miR-107, miR-181, miR-210, and miR-213) was induced in response to hypoxia, some of which were induced through a hypoxia-inducible-factor (HIF)-dependent mechanism. Bhandari et al. (50) observed hypoxia related to miRNA imbalance in cancer and verified its role in hypoxic regulation. Li et al. (51) showed that miR-21-5p has a protective effect by promoting bone matrix differentiation under hypoxic conditions. Probiotics have also been shown to regulate miRNA expression (52–54).

Muenchau et al. (55), analyzed the miRNA expression profile in human intestinal epithelial cells (IECs) in a hypoxic environment and found that miRNA-320a has a direct role in regulating IEC barrier function, suggesting proposing that a hypoxic environment in the intestinal lumen may not only mediate the regulation of tight junctions and adhesion protein expression through HIF, but also affect intestinal barrier function through miRNA-based regulation of cell-cell formation. In this study, by analyzing the sequencing results of miRNAs, we identified more than 12 600 miRNAs from all samples and 161 novo miRNAs in all groups. Subsequently, differentially expressed miRNAs in the three groups were identified. A total of 44 differentially expressed miRNAs (DEmiRNAs) were obtained, of which 13 were between HA and HAP groups, 8 were up-expressed and 5 were down-regulated. There were 22 up-regulated and 11 down-regulated DEmiRNAs between groups C and HA. There were 9 DE miRNAs between groups C and HAP, with four up-regulated and five down-regulated. These DEmiRNAs may play a role in the regulation of intestinal structure and function under hypoxia induced by *L. johnsonii* YH1136. Shi et al. (56). showed that hypoxia could inhibit the expression of miR-499-5p, and that overexpression of miR-499-5p could inhibit apoptosis induced by hypoxia/reoxygenation by targeting SOX6. In this study, the sequencing results showed that mmu-miR-499a-5p was significantly down-regulated in group HA compared to group C, which was consistent with the results of Shi et al. miR-499-5p was significantly up-regulated in the HAP group compared with the HA group, suggesting that *L. johnsonii* YH1136 may exert beneficial effects by reducing apoptosis through miR-499-5p-SOX6. MiR-122-5p was also significantly differential expressed between the HAP and HA groups, with higher expression in both groups (compared with other low expression miRNAs), which was significantly down regulated in the HAP group. Hou et al. (57) analyzed the miRNA expression profile of plasma exosomes and reported that some miRNAs, including miR-122-5p, were positively correlated with disease activity in intestinal Behçet’s syndrome.

miR-196a-1-3p and miR-3060-3p were considered important regulatory miRNAs through correlation analysis, and their target genes and potentially enriched pathways were further predicted. miR-196a-1-3p is enriched in ECM-receptor interactions, while the structural components of the ECM actively participate in intestinal inflammation (58). miR-3060-3p is enriched in the Hippo signaling pathway. A study by Yu et al. (59) showed that miR-590-5p could alleviate intestinal inflammation by targeting YAP, a component of the Hippo signaling pathway. This may explain the regulation of intestinal inflammation and injury by miR-3060-3p in response to high-altitude conditions. However, whether miR-3060-3p has similar effects requires further exploration and verification.

We further studied the role of *L. johnsonii* YH1136 supplementation in regulating miRNAs involved in the processes of intestinal injury caused by a hypoxic environment and used miRanda to predict target genes. Among them, we focused on the target genes of miRNAs differentially expresseed between groups HA and HAP. These target genes are mainly invloved in system development, signal transduction, and multicellular organism development. The Wnt signaling pathway was significantly enriched in all groups. Genetic studies have shown that the Wnt/β- catenin signaling pathway plays an important role in the proliferation of intestinal epithelial cells (60). Li et al. (61) found that the miR-155/HBP1 axis activates Wnt/β-catenin signaling pathway and induces intestinal fibrosis. In our microbiota-miRNA correlation analysis, *Lactobacillus* showed a strong positive or negative correlation with a variety of miRNAs. Therefore, we speculated that *L. johnsonii* YH1136 significantly affects the intestinal barrier by up or down regulating various miRNAs. MicroRNAs have been confirmed to remodel the composition of intestinal microbiota in several studies (62, 63), while probiotics can regulate the expression of certain miRNAs. This suggests that probiotics may not only directly affect the composition of the intestinal microbiota but may also remodel it by affecting specific miRNAs expression. In addition to the miR-196a-1-3p and miR-3060-3p, miR-485-5p, miR-7219-3p, and miR-103-2-5p were also of interest, and their target gene were predicted using the miRDB database. After screening, we found that a target gene of miR-485-5p, Pak1, the p21 activated kinase, is associated with multiple diseases (64, 65) and may play a regulatory role in intestinal inflammation induced by a high-altitude hypoxic environment. Overexpression studies of PAK1, which has been shown to have higher expression in inflammatory bowel disease, showed higher cell proliferation and reduced apoptosis when overexpressed (66). In addition, the target gene of miR-7219-3p, Adamdec1, which protects the intestine from chemical and bacterial insults that may contribute to the development of Crohn’s disease (67), was also confirmed to be downregulated by probiotics in one study (68). Likewise, Cyld, a target gene of miR-103-2-5p, plays an important regulatory role in intestinal inflammation (69). More in-depth studies are required to determine pathways that plays key regulatory roles in intestinal diseases. Rodríguez-Nogales et al. (54), pointed out that the probiotic *Saccharomyces boulardii* could play a protective role in DSS-induced colitis in mice by affecting the expression of miRNAs and the composition of the intestinal microbiota. This is similar to the protective effect of *L. johnsonii* YH1136 on the intestinal barrier in hypoxic environments.

## Conclusion

High-altitude exposure can lead to disorders in the intestinal microbiota and promote intestinal barrier impairment in mice, triggering intestinal inflammation, and endogenous pathogenic bacteria induced intestinal dysfunction. Supplementation with *L. johnsonii* YH1136 *via* gavage could be effective in alleviating these symptoms by reshaping the intestinal microbiota, reducing the abundance of pathogenic bacteria and regulating the expression of target genes in conjunction with miRNAs in the ileum. Though a number of positive results of *L. johnsonii* YH1136 were found in the present experiment, more studies in mice exposed at different altitudes should be undertaken to fully support the protective effect of such a potential probiotic strain against high altitude exposure. The specific regulatory mechanisms of miRNAs in the process of high-altitude exposure leading to intestinal barrier damage should also be demonstrated.

## Data availability statement

The data presented in the study are deposited in the NCBI SRA database, accession number PRJNA865817 (MicroRNA sequencing raw data) and PRJNA866881(16S rRNA amplicon sequencing raw data). 

## Ethics statement

The animal study was reviewed and approved by the Committee of Sichuan Agriculture University. Animal experiments were conducted in accordance with the guidelines for the feeding and use of experiment animals (approval No: SYXKchuan2019-187) approved by Committee of Sichuan agriculture University.

## Author contributions

ZW, XZ and XJ conceived the research. ZW, XZ, NS, XJ and JX performed the experiments. ZW, NS and XZ wrote the manuscript. XJ, YQ and NS analyzed the experimental data. All authors contributed to the article and approved the submitted version.

## Funding

This work is supported by the Sichuan Science and Technology Program (2021YJ0166), Scientific Development funds for Local Region from the Chinese Government in 2022 (XZ202201YD0018C), Reformation and Development Funds for Local Region Universities from the Chinese Government in 2021 (zf21003002) and Guangdong Basic and Applied Basic Research Foundation (Grant No: 2020A1515110693).

## Conflict of interest

HW was employed by the Guangzhou Beneco Biotechnology Co., Ltd.

The remaining authors declare that the research was conducted in the absence of any commercial or financial relationships that could be construed as a potential conflict of interest.

## Publisher’s note

All claims expressed in this article are solely those of the authors and do not necessarily represent those of their affiliated organizations, or those of the publisher, the editors and the reviewers. Any product that may be evaluated in this article, or claim that may be made by its manufacturer, is not guaranteed or endorsed by the publisher.

## Abbreviations

L., Lactobacillus; ASV, Amplicon Sequence Variants; IBD, Inflammation Bowel Disease; miRNA, MicroRNA; DEPC, Diethyl pyrocarbonate; DAO, Diamine oxidase; ANOVA, Analysis of Variance; LSD, Least Significant Difference; PBS, Phosphate Buffered Saline; BSA, Bovine Serum Albumin; HRP, Horseradish Peroxidase; DAB, Diaminobenzidine; DAPI, 4’,6-diamidino-2-phenylindole; PCR, Polymerase Chain Reaction; RT-qPCR, Real Time Quantitative PCR; ASV, Amplicon Sequence Variants; TNF-α, Tumor Necrosis Factor-α; TGF-β, Transforming Growth Factor-β; IL-1β (Interleukin-1β).
