# Supplementary material for: Lactobacillus johnsonii YH1136 plays a protective role against endogenous pathogenic bacteria induced intestinal dysfunction by reconstructing gut microbiota in mice exposed at high altitude
Source: Front Immunol. 2022 Oct 10;13:1007737. doi: 10.3389/fimmu.2022.1007737 (PMC9592553; doi:10.3389/fimmu.2022.1007737)
Supplement: Supplementary file 1 [file DataSheet_1.docx]

Supplementary Material

# Supplementary Figures and Tables

## Supplementary Figures
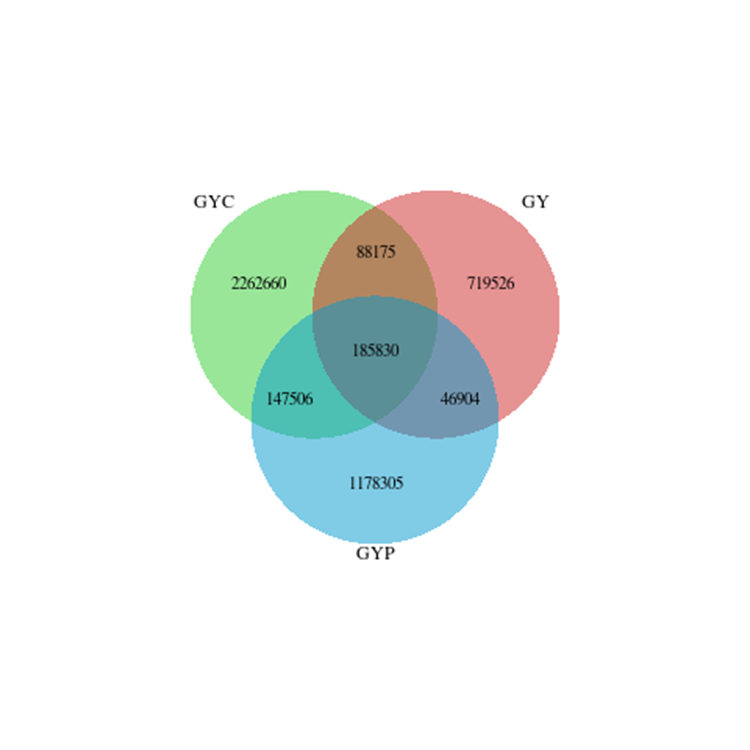


## Supplementary Figure S1. Venn graphs of all unique reads.

**
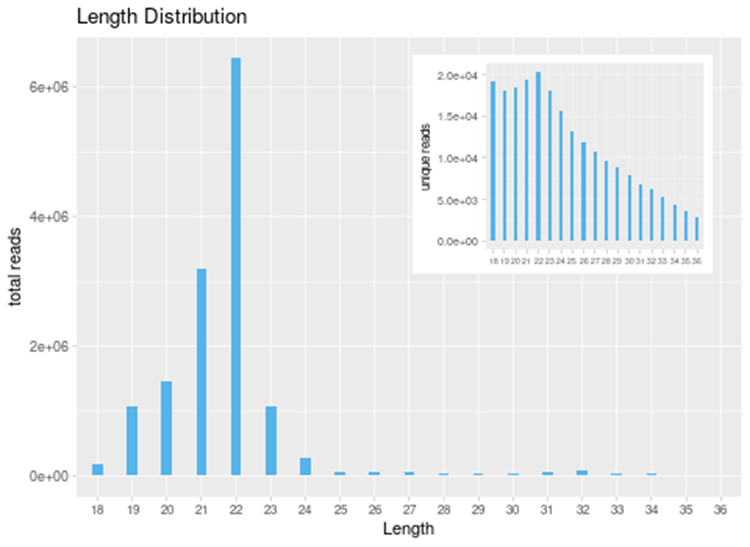
**

**Supplementary Figure S2.** **The length of total reads and unique reads (HA1).**

**
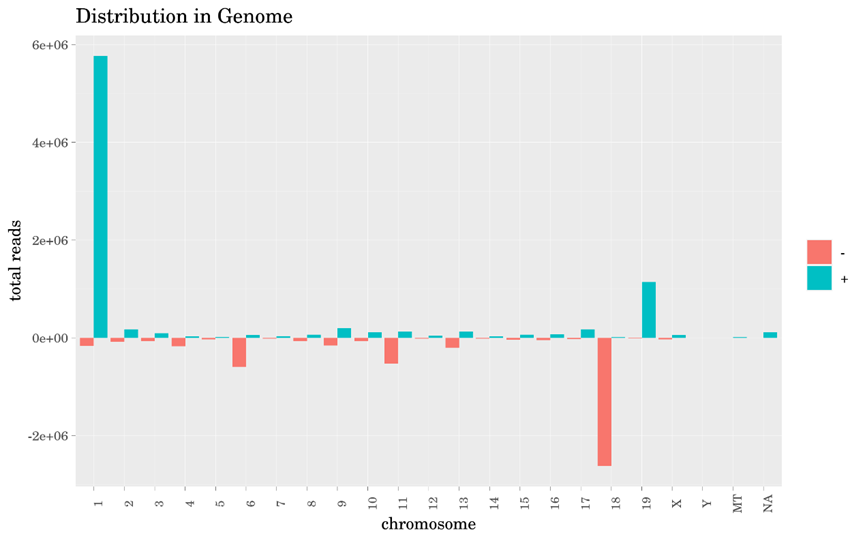
**

**Supplementary Figure S3.** **The annotation of distributions of the resulting miRNAs on chromosomes.** Taking HA1 as an example, and was mainly distributed on the DNA positive strand of chromosome 1, 19 as well as the DNA negative strand of chromosome 17.

**1.2 Supplementary Tables**

**Table S1 Specific primers for RT-PCR**

| miRNA | Sequence (5'-3') |
| --- | --- |
| mmu-miR-3473a | GTCGTATCCAGTGCAGGGTCCGAGGTATTCGCACTGGATACGACTGCTGA |
| mmu-miR-20a-3p | GTCGTATCCAGTGCAGGGTCCGAGGTATTCGCACTGGATACGACCTTTAA |
| mmu-miR-122-5p | GTCGTATCCAGTGCAGGGTCCGAGGTATTCGCACTGGATACGACCAAACA |
| mmu-miR-3060-3p | GTCGTATCCAGTGCAGGGTCCGAGGTATTCGCACTGGATACGACTGGGAG |
| mmu-miR-3102-3p | GTCGTATCCAGTGCAGGGTCCGAGGTATTCGCACTGGATACGACTGTGGG |
| mmu-miR-802-3p | GTCGTATCCAGTGCAGGGTCCGAGGTATTCGCACTGGATACGACCTGAGT |
| mmu-miR-34b-5p | GTCGTATCCAGTGCAGGGTCCGAGGTATTCGCACTGGATACGACACAATC |
| mmu-miR-5144 | GTCGTATCCAGTGCAGGGTCCGAGGTATTCGCACTGGATACGACTCTTGC |

**Table S2 Primers for qPCR**

| **miRNA** | **Sequence (5'-3')** |
| --- | --- |
| mmu-miR-3473a | Forword GCGCGTGGAGAGATGGC |
| mmu-miR-20a-3p | Forword CGCGACTGCATTACGAGCAC |
| mmu-miR-122-5p | Forword CGCGTGGAGTGTGACAATGG |
| mmu-miR-3060-3p | Forword GCGCCATAGCACAGAAGCA |
| mmu-miR-3102-3p | Forword GGAGCACCCCATTGGCTA |
| mmu-miR-802-3p | Forword CGCGACGGAGAGTCTTTGTC |
| mmu-miR-34b-5p | Forword CGCGAGGCAGTGTAATTAGCT |
| mmu-miR-5144 | Forword GCGACTGGAGACGGAAGCT |
| U6 snRNA | Forword TCGCTTCGGCAGCACATA |
|  | Reverse GGGGCCATGCTAATCTTCTC |
| Reverse Primer (Universal) | CCAGTGCAGGGTCCGAGGTA |

**Table S3** **Changes of network properties between group C and group HA**

| ID | N(C) | N(HA) | Core(HA) | Exclusive | Jaccard-score | NESH-score | DelBet |
| --- | --- | --- | --- | --- | --- | --- | --- |
| Globicatella | 2 | 2 | 1 | 2 | 0 | 2.5 | 0.909 |
| Staphylococcus | 2 | 2 | 1 | 2 | 0 | 2.5 | 0.545 |
| Agathobacter | 2 | 2 | 1 | 2 | 0 | 2.5 | 0.409 |
| Alloprevotella | 1 | 1 | 1 | 1 | 0 | 2 | 0 |
| Alloprevotella | 1 | 1 | 1 | 1 | 0 | 2 | 0 |

**Table S4 Changes of network properties between group C and group HA**

| ID | N(HA) | N(HAP) | Core(HAP) | Exclusive | Jaccard-score | NESH-score | DelBet |
| --- | --- | --- | --- | --- | --- | --- | --- |
| Corynebacterium 1 | 1 | 6 | 3 | 6 | 0 | 2.857 | 0.685 |
| Desulfovibrio | 1 | 5 | 3 | 5 | 0 | 2.667 | 0.640 |
| Bifidobacterium | 1 | 4 | 3 | 4 | 0 | 2.467 | 0.169 |
| Pseudochrobactrum | 5 | 5 | 3 | 5 | 0 | 2.333 | 0.086 |
| Prevotella 9 | 1 | 3 | 1 | 3 | 0 | 2.250 | 0.753 |

**Table S5 Driving microbes in group HA.**

| Node | Indigroup | Phylum | Genus |
| --- | --- | --- | --- |
| ASV24 | HA | Proteobacteria | Psychrobacter |
| ASV58 | HA | Proteobacteria | Oligella |
| ASV64 | HA | Proteobacteria | Oligella |
| ASV73 | HA | Actinobacteria | Corynebacterium 1 |
| ASV83 | HA | Firmicutes | Facklamia |
| ASV14 | HA | Firmicutes | Staphylococcus |
| ASV16 | HA | Firmicutes | Staphylococcus |
| ASV20 | HA | Firmicutes | Staphylococcus |
| ASV22 | HA | Proteobacteria | Psychrobacter |
| ASV60 | HA | Actinobacteria | Corynebacterium 1 |
| ASV61 | HA | Actinobacteria | Corynebacterium 1 |
| ASV80 | HA | Actinobacteria | Corynebacterium 1 |
| ASV91 | HA | Actinobacteria | Brevibacterium |

**Table S6** **KEGG pathway prediction of miR-196a-1-3p and miR-3060-3p by database Targetscan and mirPath V3**

| **miRNA** | **KEGG pathway** | **p-value** | **Genes** |
| --- | --- | --- | --- |
| miR-196a-1-3p | ECM-receptor interaction | 7.93×10^-8^ | Col24a1 |
|  | Axon guidance | 1..84×10^-3^ | Plxnc1  Pak2 |
|  | Lysine degradation | 5.64×10^-3^ | Whsc1 |
|  | Maturity onset diabetes of the young | 9.51×10^-3^ | Neurod1 |
|  | Cell adhesion molecules | 2.58×10^-2^ | Cadm1 |
| miR-3060-3p | Hippo signaling pathway | 6.25e^-12^ | Yap1 |
|  | RNA transport | 1.86×10^-3^ | Ran |
